# Ibn al-Jazzar (al-Gizar): A Renowned Tunisian Physician in General Medicine, Women’s Diseases, and Pediatrics During the Middle Ages

**DOI:** 10.7759/cureus.77274

**Published:** 2025-01-11

**Authors:** Siwar Zridi, Imen Dghim, Anis Jellad

**Affiliations:** 1 Physical Medicine and Rehabilitation, Faculty of Medicine, University of Monastir, Monastir, TUN

**Keywords:** arabic medicine, general medicine, ibn al-jazzar, islamic medical tradition, middle ages, pediatrics, tunisian physician, women’s diseases

## Abstract

Ibn al-Jazzar (al-Gizar), an influential Tunisian physician from the Middle Ages (9th-10th century), played a significant role in the development of medicine in various fields, including general medicine, women’s diseases, and pediatrics. Born in Al-Qayrawan (central region of Tunisia), he dedicated his life to serving the poor, writing extensively about both complex and accessible treatments. His most renowned work, *Zad al-Musafir wa Qut al-Hadir* (Provision for the Traveler and Nourishment for the Settled), became a medical reference widely translated into Greek, Hebrew, and Latin, influencing both Islamic and European medical practices. In addition, he wrote *Tibb al-Fuqara* (Medicine for the Poor), offering simple remedies for those unable to afford treatment. Ibn al-Jazzar also focused on women’s health, discussing menstruation disorders, fertility, and contraception in detail, often drawing from Galen's humoral theory. His pediatric contributions, including the management of children’s health and nutrition, were groundbreaking for his time. His legacy is not only preserved through his writings but also through the translation and application of his works in the West, making him a lasting figure in both Islamic and European medical traditions.

## Introduction and background

**Figure 1:**
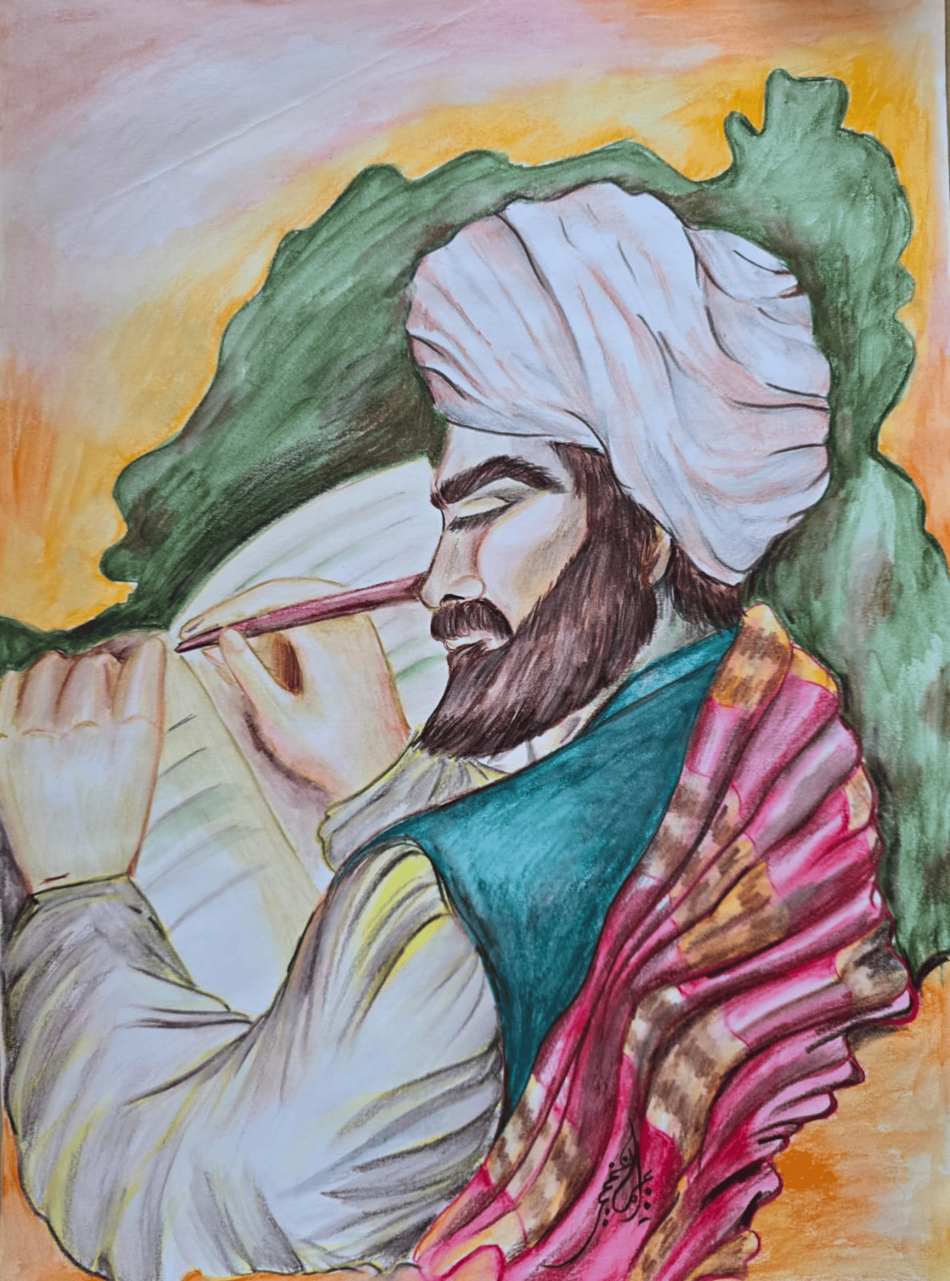
Abu Ja'far Ahmad ibn Abi Khalid ibn al-Jazzar (al-Gizar) Image credit: Dr. Imen Dghim

Abu Ja'far Ahmad ibn Abi Khalid ibn al-Jazzar, or al-Gizar (Figure 1), known as the Quayrawani, was the first Muslim physician from Africa who lived in the Middle Ages (9th-10th century AD) [1]. He was born and lived in Al-Qayrawan between 895 and 980 AD. He was a scientist in the fields of pharmacology, history, philosophy, and both adult and pediatric medicine. He played a fundamental and reliable role in the development of medicine, exploring bladder, kidney, gastrointestinal, and genital diseases [1]. One of his most famous works in the medical field was *Zad al-Musafir wa-Qut al-Hadir* (The Provisions of the Traveler and the Sustenance of the Present) [2], which is a systematic and comprehensive medical handbook. He devoted his life to the knowledge and practice of medicine to serve the poor and the needy.

## Review

Ibn al-Jazzar’s life and career

Ibn al-Jazzar was born in Al-Qayrawan, the Islamic capital of North Africa (central region of Tunisia), in the seventh century, to a family of physicians [3]. He learned medicine from his father, Ibrahim, and his paternal uncle, Abu Bakr. Besides being skilled in a variety of sciences and geography, Ibn al-Jazzar devoted himself to medicine and took all his time to examine his patients, exploring their symptoms and analyzing their urine.

Ibn al-Jazzar wrote a total of 37 books and monographs, and according to some information, there are over 40 books, but not all of them are preserved in good condition [1].

He wrote an uplifting book, *Zad al-Musafir wa Qut al-Hadir* (Provision for the Traveler and Nourishment for the Settled), which included 303 folios in MS Dresden 209. It has been a reference for many physicians and made them more productive through his own experience; proof of this is that, in the 11th century, it was translated into Greek, Hebrew in 1124, 1254, and Latin in 1124, spreading widely in the world, particularly in Europe. Ibn al-Jazzar stood by the poor, treating them for free. In this sense, he wrote another book with simpler, yet effective, treatments gathered from other wise men’s works, such as Galenus, Dioscorides, Paulus, and Hippocrates, for the needy who couldn’t afford to get treated, entitled *Tibb al-Fuqara* (Medicine for the Poor) [4]. He also wrote a book, *Siyaasat al-Sibyan wa Tadbirhom* (The Governance of Children and Their Management), in pediatric medicine.

He stayed away from those in power and wealth, dedicating his free time to worship and spiritual practice. Ibn al-Jazzar was not only a brilliant physician but also a good writer, and he had a great impact on physicians in the Golden Age of Islam. Later evidence of this can be found in references to his work in the writings of other famous physicians, such as Al-Razi, Ibn Sina, Al-Akhaweini, and Al-Zahrawi [1].

Medicine for the Poor and Destitute

The most famous book, *The Provisions of the Traveler* *and* *the Sustenance of the Present*, was written by Ibn al-Jazzar. It served many physicians throughout the years. Unfortunately, it was difficult for the poor to benefit from it due to its complexity and the lack of means to obtain the recommended drugs or food potions. Ibn al-Jazzar gave specific attention to the needs of the poor, who couldn’t afford medical care. *Tibb al-Fuqara wa’l-Masakin* (Medicine for the Poor and Destitute) is a small monograph written in the 10th century, and it has been a popular book throughout the Middle Ages in both Islamic and Western Latin literature. In his book, the first chapter starts with a recipe for headaches, and the last chapter, number 25b, treats podagra or gout.

Although it’s small in size, it served the needy with its numerous simple potions and traditional medicinal recipes, using affordable elements that are available almost anywhere and at any time of need, so that they may be cured for free. He even gave multiple choices of treatments for each disease or discomfort.

His book was assumed to have been translated by the Latin West into a text entitled *Liber Pauperum* in the 10th century by Hayyim ben Judah Ibn Musah, a physician known for his professional ability in the service of kings and nobles for 40 years, who found this book to be useful after testing several of its remedies.

He was the one who introduced the use of animal parts and decrements into therapeutics: treating alopecia, otitis, conjunctivitis, croup, colic, and podagra [5]. Because the poor were living in squalor and dirt, they were more susceptible to being attacked by snakes, scorpions, and all sorts of vermin. Ibn al-Jazzar also details remedies to treat all kinds of stings and venomous bites. 

Women’s diseases

Ibn al-Jazzar, a devoted follower of Galen, discussed women’s diseases in his book *Zad al-Musafir*. He explored various topics, including menstruation retention and sexual diseases affecting both men and women across 20 chapters. Additionally, he addressed childbirth difficulties, recommending the use of a tone or a cyclamen hung on the thigh of the woman to assist in the extraction of the placenta [2]. In his other work, *Tibb al-Fuqara*, he examined fertility and contraception and detailed multiple natural methods and magical remedies that women could use either to prevent conception or stimulate it.

Women’s diseases were discussed in chapters 9 to 18 of the sixth book of *Zad al-Musafir*. The central topic of chapter 9 is the retention of menstrual blood, known as amenorrhea. Ibn al-Jazzar divided amenorrhea into two types, as Galen did: natural and accidental [1]. Natural amenorrhea occurs around the ages of 50 and 60, marking menopause. He also discussed early menopause, which can happen as early as age 35, highlighting characteristics of women who may be prone to this condition. He detailed the symptoms of amenorrhea based on Galen, including lack of appetite, nausea, and cravings for substances like charcoal and earth, which may also indicate pregnancy [1]. This topic is discussed in more depth in chapter 15, which focuses on the regimen of pregnant women. One of the causes of amenorrhea detailed in Ibn al-Jazzar’s work is psychological amenorrhea, which can arise from sorrow, anxiety, anger, and fear. In chapter 10, he discussed hypermenorrhea, including its symptoms, causes, and treatment options.

Ibn al-Jazzar relied on the humoral theory of medicine, a concept deeply rooted in Galen’s work, which emphasizes the importance of balancing bodily fluids (humors) for maintaining health. Regarding dysmenorrhea, he advocated for venesection, applying it to the upper part of the body to treat hypermenorrhea and to the lower limbs to treat amenorrhea [1].

In chapter 11, Ibn al-Jazzar examined a common psychiatric condition known as hysterical conversion, referred to as hysterical suffocation, which predominantly affects women. Symptoms of this disorder include coldness, fainting, weak pulse, and convulsion-like symptoms. According to the author, this psychological ailment primarily impacts virgins and widows [1].

Furthermore, Ibn al-Jazzar was well-acquainted with tumors of the uterus and cervix in women. His access to translated Greek texts enabled him to incorporate Greek medical theory, including diagnostic and treatment methods, into his work. His influence from Paul of Aegina, particularly in his *Epitome of Medicine*, is evident in his comprehensive understanding of gynecological tumors, covering risk factors, symptoms, and treatment. This topic is significantly addressed in chapter 13 [6].

Pediatrics

Ibn al-Jazzar wrote a whole book dedicated to children entitled *Siyaasat al-Sibyan wa Tadbirihim* (The Management of Children), comprising 22 chapters. He discussed various health issues that may compromise children’s development, illnesses, and nutrition.

He paid such detailed attention to children’s well-being, elaborating treatments to soothe babies' teething using henna and licorice, treating gastrointestinal diseases including vomiting, diarrhea, and fungal and parasitic infections using different types of plants and natural ingredients [7].

In his book, he also devoted a whole chapter to a discussion of bladder stones, including their etiology, sex incidence, symptoms, and signs [8]. He also performed the first meatotomy, used clysters as preparation for lithotomy, and crushed large bladder stones [8].

## Conclusions

Ibn al-Jazzar stands as a pivotal figure in the Arabic history of medicine. His innovative contribution, especially in general medicine, women’s diseases, and pediatrics, was a great cornerstone in the development of healthcare throughout the decades. His medical books were translated into multiple languages, leading to a lasting impact in both Europe and the Arabic world. His legacy is engraved in memory and will continue to inspire medical researchers and students in both Western and Eastern medical traditions.
